# Amino acid digestibility in low-fat distillers dried grains with solubles fed to growing pigs

**DOI:** 10.1186/2049-1891-5-27

**Published:** 2014-05-13

**Authors:** Shelby Marie Curry, Diego Mario David Labadan Navarro, Ferdinando Nielsen Almeida, Juliana Abranches Soares Almeida, Hans Henrik Stein

**Affiliations:** 1 Department of Animal Sciences, University of Illinois, Urbana 61801, USA

**Keywords:** Amino acid digestibility, Distillers dried grains with solubles, Pigs

## Abstract

The objective of this experiment was to determine the standardized ileal digestibility (SID) of amino acids (AA) in 3 sources of distillers dried grains with solubles (DDGS) with different concentrations of fat. Twelve growing barrows (initial body weight: 76.1 ± 6.2 kg) were randomly allotted to a replicated 6 × 4 Youden square design with 6 diets and 4 periods. The fat content of the 3 sources of DDGS were 11.5, 7.5, and 6.9% respectively. Diets contained 60% DDGS and fat concentration of the diets were 7.5, 5.2, and 5.2%, respectively. Two additional diets containing the 2 sources of DDGS with 7.5 and 6.9% fat were also formulated, and corn oil was added to these diets to increase the concentration of fat in the diets to levels that were calculated to be similar to the diet containing conventional DDGS with 11.5% fat. A N-free diet was also formulated to calculate endogenous losses of crude protein (CP) and AA from the pigs. Pigs were fed experimental diets during four 7-d periods. The first 5 d of each period were an adaptation period and ileal digesta were collected on d 6 and 7 of each period. The apparent ileal digestibililty (AID) and SID of CP and all indispensable AA, except AID Pro and SID of Trp, were greater (*P* < 0.01) in conventional DDGS than in the 2 sources of DDGS with reduced fat. Adding oil to the diets containing the 2 sources of DDGS with reduced fat did not consistently increase SID of AA. In conclusion, conventional DDGS has greater SID values for most AA compared with DDGS that contains less fat and inclusion of additional oil to diets containing low-fat DDGS does not increase AID or SID of AA. The lower AA digestibility in low-fat DDGS could not be overcome by the inclusion of additional fat to the diets.

## Background

Distillers co-products have been used in swine diets for more than 50 years, but the increase in ethanol production in the last few decades has made distillers dried grains an available and attractive ingredient to use in swine diets [1]. Conventional distillers dried grains with solubles (DDGS) contains approximately 27% CP, 10% fat, 9% acid detergent fibre (ADF), and 25% neutral detergent fibre (NDF) [1-3]. However, new technologies have been developed and implemented to remove fat from DDGS to be able to market the oil for biodiesel production or other uses. If oil is removed by centrifugation of the solubles before solubles are added to the distilled grains, then a low-fat DDGS is produced. Low-fat DDGS contains 6 to 9% oil [3]. If oil is extracted from DDGS using a solvent extraction process, de-oiled DDGS is produced [3]. De-oiled DDGS contains less than 4% oil, and therefore, contains less energy than conventional DDGS [4], which may affect its economic value and inclusion level [5]. Oil removal improves handling issues commonly encountered when using DDGS in pig diets such as challenges with flowability [4].

The DE and ME of de-oiled and low-fat DDGS [4,6] are less than the DE and ME of conventional DDGS. There is, however, limited information about the digestibility of AA in low-fat DDGS, but increased concentrations of dietary fat may increase the digestibility of AA in soybean meal and in mixed corn-soybean meal-DDGS diets [7-10]. It is, therefore, possible that the digestibility of AA is also influenced by the concentration of fat in DDGS. The objective of this research was to determine if the concentration of fat in DDGS and diets containing DDGS affects the apparent ileal digestibility (AID) or standardized ileal digestibility (SID) of CP and AA fed to growing pigs.

## Materials and methods

The Institutional Animal Care and Use Committee at the University of Illinois reviewed and approved the protocol for this experiment.

### Animals, housing, experimental design, and diets

A total of 12 growing barrows (Genetiporc, Alexandria, MN) with an initial body weight (BW) of 76.1 ± 6.2 kg were randomly allotted to a replicated 6 × 4 Youden square design with 6 diets and four 7-d periods. Pigs had a cannula surgically installed in the distal ileum to allow for collection of ileal digesta [11]. Each pig was individually housed in a 1.2 m × 1.5 m that was equipped with a feeder and a nipple drinker. Pens were in a temperature-controlled barn with a propane heater and forced air fans. Each pen has smooth sidings and a fully slatted Tri-bar steel floor.

Three sources of DDGS were produced by Poet Nutrition, Sioux Falls, SD (Table 1). The 3 sources of DDGS were produced at the same facility on 3 consecutive days in the fall of 2011. The corn that was used on these 3 days was from a common source. The DDGS that was produced on the first day consisted of distillers dried grains and all the solubles. However, fat was skimmed off the solubles that were added to the distiller dried grains on d 2 and 3, resulting in production of 2 sources of low-fat DDGS. The conventional DDGS contained 11.5% fat, and the two sources of low-fat DDGS contained 7.5% and 6.9% fat, respectively. Six diets were formulated (Tables 2 and 3). Three diets that contained 60% of each source of DDGS were formulated and these diets contained 7.5, 5.2, and 5.2% fat, respectively. Two additional diets were also formulated and these diets also contained the 2 sources of low-fat DDGS, but corn oil was added to these diets with the intent to bring the concentration of fat in the diets to the same level as in the diet with the conventional DDGS. A N-free diet was used to measure endogenous losses of CP and AA from the pigs. Chromic oxide (0.4%) was included in all the diets as an inert marker. Vitamins and minerals were included in all diets to meet requirements [12].

**Table 1:** **Analyzed nutrient composition of ingredients (as-fed basis)**^**1**^

	**Ingredients**^**2**^		
**Item**	**Conventional DDGS**	**Low- fat DDGS, 7.5% fat**	**Low-fat DDGS, 6.9% fat**
DM, %	90.61	89.04	87.48
CP, %	25.73	28.03	27.93
AEE^3^, %	11.47	7.51	6.86
ADF, %	7.78	9.27	8.82
NDF, %	28.64	30.74	29.87
Starch, %	1.50	1.16	1.33
Ca, %	0.07	0.06	0.06
P, %	0.90	0.87	0.92
Indispensable AA, %			
Arg	1.22	1.22	1.24
His	0.69	0.75	0.71
Ile	1.03	1.12	1.06
Leu	2.79	3.17	3.07
Lys	0.91	0.91	0.88
Met	0.52	0.59	0.55
Phe	1.25	1.36	1.35
Thr	0.97	1.02	1.02
Trp	0.21	0.20	0.20
Val	1.30	1.43	1.33
Dispensable AA, %			
Ala	1.72	1.93	1.86
Asp	1.59	1.73	1.68
Cys	0.62	0.62	0.63
Glu	3.28	4.01	3.81
Gly	1.05	1.10	1.06
Pro	1.91	2.08	2.17
Ser	1.07	1.13	1.18
Tyr	1.01	1.09	1.09

^1^Ingredients were obtained from Poet Nutrition, Sioux Falls, SD.

^2^DDGS = distillers dried grains with solubles.

^3^AEE = acid hydrolyzed ether extract.

**Table 2:** Ingredient composition of experimental diets, as-fed basis

	**Diet**^**1**^					
**Ingredient, %**	**Conventional DDGS**	**Low-fat DDGS, 7.5% fat**	**Low-fat DDGS, 6.9% fat**	**Low-fat DDGS, 7.5% fat with oil**	**Low-fat DDGS, 6.9% fat with oil**	**N-free**
Cornstarch	27.60	27.60	27.60	28.20	28.25	69.00
Conventional DDGS	60.00	-	-	-	-	-
Low-fat DDGS-1	-	60.00	-	58.20	-	-
Low-fat DDGS-2	-	-	60.00	-	57.20	-
Corn oil	-	-	-	1.20	2.15	4.00
Sugar	10.00	10.00	10.00	10.00	10.00	20.00
Chromic oxide	0.40	0.40	0.40	0.40	0.40	0.40
Limestone	1.30	1.30	1.30	1.30	1.30	0.60
Salt	0.40	0.40	0.40	0.40	0.40	0.40
Vitamin-mineral premix^2^	0.30	0.30	0.30	0.30	0.30	0.30
Cellulose^3^	-	-	-	-	-	4.00
Dicalcium Phosphate	-	-	-	-	-	1.30

^1^DDGS = distillers dried grains with solubles.

^2^The vitamin-micromineral premix provided the following quantities of vitamins and micro minerals per kilogram of the complete diet: vitamin A as retinyl acetate, 11,128 IU; vitamin D_3_ as cholecalciferol, 2,204 IU; vitamin E as DL-alpha tocopheryl acetate, 66 IU; vitamin K as menadione nicotinamide bisulfate, 1.42 mg; thiamin as thiamine mononitrate, 0.24 mg; riboflavin, 6.58 mg; pyridoxine as pyridoxine hydrocloride, 0.24 mg; vitamin B_12_, 0.03 mg; D-pantothenic acid as D-calcium pantothenate, 23.5 mg; niacin as nicotinamide and nicotinic acid, 44 mg; folic acid, 1.58 mg; biotin, 0.44 mg; Cu, 10 mg as copper sulfate; Fe, 125 mg as iron sulfate; I, 1.26 mg as potassium iodate; Mn, 60 mg as manganese sulfate; Se, 0.3 mg as sodium selenite; and Zn, 100 mg as zinc oxide.

^3^Solka Floc. Fiber Sales and Development Corp., Urbana, OH.

**Table 3:** Analyzed nutrient composition of experimental diets, as-fed basis

	**Diet**^**1**^					
**Item**	**Conventional DDGS**	**Low-fat DDGS, 7.5% fat**	**Low-fat DDGS, 6.9% fat**	**Low-fat DDGS, 7.5% fat with oil**	**Low-fat DDGS, 6.9% fat with oil**	**N-free**
CP, %	15.62	16.49	16.59	16.01	15.65	0.28
DM, %	92.26	90.99	89.57	90.20	87.67	91.93
AEE ^2^, %	7.47	5.20	5.15	6.21	6.95	4.07
Indispensable AA, %						
Arg	0.73	0.72	0.75	0.72	0.70	0.01
His	0.43	0.46	0.46	0.45	0.41	0.00
Ile	0.57	0.62	0.61	0.60	0.55	0.01
Leu	1.69	1.91	1.88	1.88	1.74	0.02
Lys	0.51	0.51	0.51	0.50	0.47	0.01
Met	0.31	0.35	0.33	0.34	0.31	0.00
Phe	0.73	0.81	0.80	0.79	0.74	0.01
Thr	0.58	0.62	0.63	0.63	0.60	0.01
Trp	0.13	0.12	0.13	0.12	0.12	<0.04
Val	0.81	0.88	0.87	0.84	0.77	0.01
Dispensable AA, %						
Ala	1.08	1.19	1.18	1.17	1.09	0.01
Asp	1.01	1.08	1.08	1.06	1.00	0.01
Cys	0.29	0.32	0.31	0.32	0.28	0.00
Glu	2.38	2.62	2.58	2.56	2.40	0.03
Gly	0.64	0.67	0.68	0.66	0.62	0.01
Pro	1.24	1.40	1.38	1.38	1.30	0.06
Ser	0.62	0.67	0.69	0.71	0.68	0.01
Tyr	0.54	0.58	0.58	0.59	0.56	0.01

^1^DDGS = distillers dried grain with solubles.

^2^AEE = acid hydrolyzed ether extract.

### Feeding and sample collections

Pigs were fed at a level of 3 times the maintenance energy requirement (i.e., 106 kcal ME per kgBW^0.75^; [12]) and the daily feed allotments were divided into 2 equal meals. Amount of feed supplied was recorded daily and pig weights were recorded at the beginning of the experiment and at the end of each 7-d period. The initial 5 d of each period was considered a diet adaptation period. Ileal digesta were collected on d 6 and 7 for 8 h as previously described [13]. All samples were stored at −20°C immediately after collection to avoid bacterial degradation of the AA. At the conclusion of the experiment, samples were thawed and mixed within animal and diet and a sub-sample was collected and analyzed.

### Chemical analysis

A sample from each ingredient and each diet was collected at the time of diet mixing. Ileal digesta samples were lyophilized and ground prior to chemical analysis. Samples from diets and digesta were analyzed for dry matter (DM; Method 930.15; [14]), chromium (Method 990.08; [14]), CP (Method 990.03; [14]), and AA (Method 982.30 E (a, b, c); [14]). Prior to AA analysis, samples were hydrolyzed with 6 mol/L HCl for 24 h at 110°C (method 982.30 E(a); [14]). Methionine and Cys were determined as Met sulfone and cysteic acid after cold performic acid oxidation overnight before hydrolysis (method 982.30 E(b); [14]). Tryptophan was determined after NaOH hydrolysis for 22 h at 110°C (method 982.30 E(c); [14]). Each ingredient was also analyzed for DM, CP, AA, ADF (Method 973.18; [14]), NDF [15], for Ca and P by inductively coupled plasma spectroscopy (Method 985.01; [14]), and for starch using the glucoamylase procedure (Method 979.10; [14]). Each ingredient and all diets were also analyzed for total fat by acid hydrolysis using 3 *N* HCl [16] followed by crude fat extraction using petroleum ether (Method 2003.06; [14]) on a Soxtec 2050 automated analyzer (FOSS North America, Eden Prairie, MN).

### Calculations and statistical analysis

Basal endogenous losses of CP and AA were determined from pigs after feeding the N-free diet. Values for AID, endogenous losses, and SID of CP and AA were calculated as previously described [17]. The MIXED procedure of SAS was used to analyze the data (SAS Inst. Inc., Cary, NC). The model included diet as a fixed effect whereas pig and period were included as random effects. A pig was used as the experimental unit. The UNIVARIATE procedure of SAS was used to determine if there were any outliers. However, no outliers were identified. An observation was considered an outlier if the value was more than 3 standard deviations away from the grand mean. The LSMeans statement was used to calculate mean values for each diet and the PDIFF option was used to separate means. Moreover, an orthogonal contrast was conducted to verify if the addition of oil to low-fat DDGS diets increases AA digestibility of low-fat DDGS to the same level as conventional DDGS. The contrast was performed by comparing conventional DDGS and the 2 low-fat DDGS sources with addition of supplemental oil. For all analyses, an alpha value of 0.05 was used to determine significance among means.

## Results

The AID for CP and all indispensable AA was greater (*P* < 0.01) in conventional DDGS than in the 2 low-fat sources of DDGS, whereas no differences between the 2 low-fat sources of DDGS were observed (Table 4). The mean AID of indispensable AA and the mean of dispensable AA were also greater (*P* < 0.01) in conventional DDGS than in the 2 low-fat sources of DDGS. Addition of oil to the diets containing the low-fat DDGS did not increase AID values for CP or AA.

**Table 4:** **Apparent ileal digestibilitly of CP and AA in distillers dried grains with solubles (DDGS) fed to pigs**^**1**^

	**Ingredients**							**Additional oil**^**2**^	
**Item**	**Conventional DDGS**	**Low-fat DDGS, 7.5% fat**	**Low-fat DDGS, 6.9% fat**	**Low-fat DDGS, 7.5% fat with oil**	**Low-fat DDGS, 6.9% fat with oil**	**SEM**	***P*-value**	**SEM**	***P*-value**
CP, %	71.8^a^	64.6^b^	66.1^b^	66.9^b^	68.0^b^	1.26	<0.01	1.54	<0.01
Indispensable AA, %									
Arg	81.8^a^	75.0^b^	76.9^b^	77.3^b^	75.5^b^	0.93	<0.01	5.88	<0.01
His	78.0^a^	70.8^b^	71.9^b^	72.9^b^	70.3^b^	1.12	<0.01	1.08	<0.01
Ile	75.6^a^	69.1^b^	69.3^b^	71.2^b^	67.8^b^	0.95	<0.01	1.00	<0.01
Leu	85.4^a^	81.4^b^	80.2^b^	82.8^b^	81.2^b^	0.76	<0.01	0.69	<0.01
Lys	62.2^a^	50.8^b^	56.1^b^	56.9^b^	51.4^b^	1.91	<0.01	1.51	<0.01
Met	85.8^a^	82.8^b^	81.5^b^	83.7^b^	82.8^b^	0.68	<0.01	0.63	<0.01
Phe	81.5^a^	77.2^b^	76.8^b^	78.5^b^	76.8^b^	0.75	<0.01	0.71	<0.01
Thr	65.8^a^	59.9^b^	61.4^b^	63.6^b^	61.2^b^	1.30	<0.01	1.18	<0.01
Trp	76.5^a^	70.8^b^	74.7^b^	74.1^b^	71.3^b^	1.23	<0.01	1.36	0.01
Val	75.7^a^	69.8^b^	70.3^b^	71.4^b^	68.2^b^	0.95	<0.01	0.99	<0.01
Mean	78.3^a^	72.8^b^	73.2^b^	74.9^b^	72.5^b^	0.85	<0.01	0.84	<0.01
Dispensable AA, %									
Ala	80.7^a^	76.5^b^	75.8^b^	77.9^b^	76.2^b^	0.89	<0.01	0.84	<0.01
Asp	67.7^a^	62.3^b^	63.3^b^	65.1^b^	61.4^b^	1.13	<0.01	1.16	<0.01
Cys	71.7^a^	63.4^b^	64.8^b^	67.1^b^	63.0^b^	1.60	<0.01	1.41	<0.01
Glu	82.4^a^	77.4^b^	76.8^b^	78.7^b^	77.3^b^	0.96	<0.01	0.85	<0.01
Gly	58.6^a^	48.5^b^	51.0^b^	51.6^b^	47.4^b^	2.13	<0.01	2.24	<0.01
Pro	64.3	57.5	59.7	59.6	57.4	4.69	0.37	3.16	0.08
Ser	73.4^a^	69.4^b^	70.0^b^	72.8^b^	72.0^b^	1.23	<0.01	1.02	0.34
Tyr	82.3^a^	77.7^b^	77.6^b^	79.9^b^	78.5^b^	0.72	<0.01	0.72	<0.01
Mean	74.3^a^	68.7^b^	69.0^b^	70.8^b^	68.7^b^	1.39	<0.01	1.30	<0.01
Total AA	76.1^a^	70.5^b^	70.9^b^	72.7^b^	70.4^b^	1.10	<0.01	1.08	<0.01

^a,b^Means without a common superscript in the same row differ; pairwise comparison.

^1^Data are means of 8 observations.

^2^Orthogonal contrast comparing conventional DDGS and the 2 sources of low-fat DDGS with additional oil.

The SID of CP and all indispensable AA except Trp was greater in conventional DDGS (*P* < 0.01) than in the 2 sources of low-fat DDGS (Table 5). The SID of Lys was greater (*P* < 0.01) in one of the sources of low-fat DDGS than in the other source. However, if fat was added, there was an increase in SID of Lys in the DDGS with 7.5% fat content, but a reduction in SID of Lys in the low-fat DDGS with 6.9% fat content. For Trp, the SID in conventional DDGS did not differ from that of the low-fat DDGS (DDGS, 6.9% fat) without added oil and low fat DDGS (DDGS, 7.5%) with added oil, but the SID Trp of conventional DDGS was greater (*P* < 0.05) than in the low-fat DDGS (DDGS, 7.5% fat) without added oil and low-fat DDGS (DDGS, 6.9%) with added oil. For all AA except Ser, the AID and SID of low-fat DDGS with added oil was smaller (*P* < 0.05) than AID and SID of conventional DDGS, indicating that the addition of oil did not increase AID and SID of low-fat DDGS to the same level as AID and SID of conventional DDGS (Table 5). The SID of all dispensable AA was greater (*P* < 0.01) in conventional DDGS than in the 2 sources of low-fat DDGS. Addition of oil to the diets containing low-fat DDGS did not increase SID values.

**Table 5:** **Standardized ileal digestibility of CP and AA in distillers dried grains with solubles (DDGS) fed to pigs**^**1**^

	**Ingredients**							**Additional oil**^**2**^	
**Item**	**Conventional DDGS**	**Low-fat DDGS, 7.5% fat**	**Low-fat DDGS, 6.9% fat**	**Low-fat DDGS, 7.5% fat with oil**	**Low-fat DDGS, 6.9% fat with oil**	**SEM**	***P*-value**	**SEM**	***P*-value**
CP, %	79.8^a^	72.8^b^	73.6^b^	74.6^b^	75.3^b^	1.22	< 0.01	1.29	<0.01
Indispensable AA, %									
Arg	87.7^a^	81.0^c^	82.5^bc^	83.2^b^	81.4^bc^	0.93	< 0.01	0.92	<0.01
His	80.9^a^	73.5^b^	74.6^b^	75.6^b^	73.3^b^	1.12	< 0.01	1.08	<0.01
Ile	79.8^a^	72.9^bc^	73.1^bc^	75.2^b^	71.9^c^	0.95	< 0.01	1.00	<0.01
Leu	87.7^a^	83.4^bc^	82.2^c^	84.8^b^	83.3^bc^	0.76	< 0.01	0.69	<0.01
Lys	67.9^a^	56.4^c^	61.7^b^	62.6^b^	57.3^c^	1.91	< 0.01	1.51	<0.01
Met	88.1^a^	84.8^bc^	83.6^c^	85.8^b^	85.0^bc^	0.68	< 0.01	0.63	<0.01
Phe	84.9^a^	80.3^bc^	79.8^c^	81.6^b^	80.0^bc^	0.75	< 0.01	0.71	<0.01
Thr	73.4^a^	66.9^c^	68.2^bc^	70.4^b^	68.2^bc^	1.30	< 0.01	1.18	<0.01
Trp	83.1^a^	77.8^c^	81.1^ab^	81.0^abc^	78.0^bc^	1.23	< 0.05	1.36	0.02
Val	80.5^a^	74.2^bc^	74.6^bc^	75.9^b^	73.0^c^	0.95	< 0.01	0.99	<0.01
Mean	82.5^a^	76.7^c^	77.0^bc^	78.8^b^	76.6^c^	0.85	< 0.01	0.85	<0.01
Dispensable AA, %									
Ala	85.4^a^	80.6^bc^	79.9^c^	82.1^b^	80.6^bc^	0.89	< 0.01	0.84	<0.01
Asp	73.6^a^	67.8^c^	68.7^bc^	70.6^b^	67.1^c^	1.13	< 0.01	1.16	<0.01
Cys	76.0^a^	67.3^c^	68.8^bc^	70.9^b^	67.3^c^	1.60	< 0.01	1.41	<0.01
Glu	85.4^a^	80.1^b^	79.5^b^	81.4^b^	80.2^b^	0.96	< 0.01	0.85	<0.01
Gly	75.9^a^	64.8^b^	66.8^b^	68.0^b^	64.3^b^	2.13	< 0.01	2.24	<0.01
Pro	98.5^a^	87.4^b^	89.5^b^	89.7^b^	88.4^b^	4.70	< 0.05	3.16	<0.01
Ser	78.8^a^	74.4^c^	74.7^bc^	77.4^a^	76.7^ab^	1.22	< 0.01	1.02	0.10
Tyr	85.7^a^	80.9^c^	80.7^c^	83.0^b^	81.7^bc^	0.72	< 0.01	0.71	<0.01
Mean	84.3^a^	77.7^b^	78.0^b^	79.8^b^	78.0^b^	1.39	< 0.01	1.30	<0.01
Total AA	83.5^a^	77.2^b^	77.5^b^	79.4^b^	77.4^b^	1.10	< 0.01	1.08	<0.01

^a,b^Means without a common superscript in the same row differ; pairwise comparison.

^1^Data are means of 8 observations. Values for standardized ileal digestibility were calculated by correcting apparent ileal digestibility values for basal endogenous losses (g/kg of DMI), which were determined by feeding pigs a N-free diet; CP, 13.84; Arg, 0.47; His, 0.14; Ile, 0.26; Leu, 0.42; Lys, 0.32; Met, 0.08; Phe, 0.27; Thr, 0.48; Trp, 0.09; Val, 0.39; Ala, 0.55; Asp, 0.65; Cys, 0.14; Glu, 0.78; Gly, 1.20; Pro, 4.60; Ser, 0.37; Tyr, 0.20.

^2^Orthogonal contrast comparing conventional DDGS and the 2 sources of low-fat DDGS with additional oil.

## Discussion

Distillers dried grains with solubles is a co-product from the dry-grind processing of corn and has been used in swine diets for many years. The use of DDGS in swine diets has increased because it is affordable as well as high in energy, AA, and digestible P [1,2,18]. However, the drying process of DDGS may cause heat damage to the ingredient because it involves high temperatures and moisture, and these conditions are favorable for initiating the Maillard reaction, which reduces AA concentration and digestibility [19]. The Lys:CP indicates heat damage, and a ratio greater than 2.8% is desirable in DDGS and implies no heat damage [20]. Samples used in this experiment had Lys:CP of at least 3.15% indicating that the 3 sources of DDGS used in this experiment were not heat damaged.

In recent years, some ethanol plants have centrifuged the solules that are produced to extract oil, which may be sold to the biodiesel industry [5]. The result of the centrifugation is a reduction in fat concentration from 10% in conventional DDGS to 6 to 9% in low-fat DDGS [3]. The 2 sources of DDGS used in this experiment that were produced after the solubles had the oil removed contained 7.5 and 6.9% fat respectively, and these sources of DDGS were classified as low-fat DDGS, according to the definition by NRC [3].

The starch level in the 3 sources of DDGS used in this experiment was less than previously reported [1], indicating that the fermentation process in the ethanol plant was very efficient. The ADF levels of DDGS used in this experiment were also less than reported by NRC [3]. The CP, fat, and AA levels were, however, in agreement with previous values reported [3]. The values for SID of AA and CP for the low-fat DDGS were within the range of expected values [3]. However, SID of CP, Arg, Met, Trp, Ala, Asp, and Ser of DDGS were greater than previously reported [3]. Moreover, the SID of the other AA are in the upper limit of the range reported [3]. It is possible that the apparent reduction in SID values in low-fat DDGS may be due to the higher coefficients of digestibility of the conventional DDGS used in this experiment compared with previously reported values [3].

There is limited information about the AA digestibility of low-fat DDGS or effects of additional dietary fat on AID and SID of low-fat DDGS. However, data on the effect of low-fat DDGS on digestible energy (DE) and metabolizable energy (ME) indicate that dietary fat is not always a good predictor of ME for swine [5,6,10]. Different estimates for DE and ME have been reported for different sources of DDGS even if fat concentration was similar [4,6].

The AID of AA in nursery and growing pigs is increased by the inclusion of additional fat to the diet [7,21]. The increase in dietary fat delays gastric emptying [22] and although the length of the fatty acid can be different among different sources of vegetable oil, the impact of fatty acids on gastric emptying is similar regardless of chain length [22]. The slower gastric emptying may result in slower rate of passage of the diet, causing an increase in the time of exposure of feed to proteolytic enzymes, thus providing longer time for peptides and AA to be digested and absorbed, and increase in AID of AA [7]. The addition of oil to diets fed to growing pigs increase not only the AID but also the SID of AA [9,10].

Results of this experiment indicate that the addition of dietary fat to diets containing low-fat DDGS fed to growing pigs did not improve the AID or SID of AA. These results are not in agreement with previous data [7,9]. However, the difference in fat levels between diets without or with added fat were much greater (4.0 vs. 7.5%, 0.24 vs. 6.7%) in other experiments [7,9] compared with the differences observed in the present experiment (5.2 vs. 6.2%, or 5.2 vs. 7.0%). Moreover, the fat level of the low-fat DDGS diets were relatively high and similar, and that could be the reason additional inclusion of fat did not result in an increase in SID of AA.

## Conclusions

Results of this experiment indicate that removal of oil may result in reduced AID and SID of AA in DDGS, and that the 2 sources of DDGS used in this experiment had greater SID of AA compared with previously reported values. The AID and SID of AA in low-fat DDGS were not improved by the inclusion of additional fat in the diet.

## Abbreviations

AA: Amino acids; ADF: Acid detergent fibre; AID: Apparent ileal digestibility; BW: Body weight; CP: Crude protein; DDGS: Distillers dried grains with solubles; DE: Digestible energy; DM: Dry matter; ME: Metabolizable energy; NDF: Neutral detergent fibre; SID: Standardized ileal digestibility.

## Competing interests

The authors declare that they have no competing interests.

## Authors’ contributions

All authors read and approved the final manuscript.
